# Effects of Aerobic Exercise on Cardiopulmonary Function in Postoperative Patients with Congenital Heart Disease: A Meta-analysis

**DOI:** 10.31083/j.rcm2508296

**Published:** 2024-08-21

**Authors:** Xiaozhen Guo, Yanran Si, Hairong Liu, Ling Yu

**Affiliations:** ^1^Department of Physical Education, Tongji University, 200092 Shanghai, China; ^2^Physical Education Department of Shanghai International Studies University, 201620 Shanghai, China; ^3^School of Sports and Health of Shanghai Lixin University of Accounting and Finance, 201209 Shanghai, China

**Keywords:** aerobic exercise, congenital heart disease, Peak VO_2_, cardiopulmonary function, meta-analysis

## Abstract

**Background:**

This meta-analysis aimed to evaluate the impact of aerobic exercise on Peak VO_2_ (Oxygen Consumption) in postoperative patients with congenital heart disease (CHD). Besides this, we also tried to discover whether the improvement was influenced by patient ages, modes of supervision, types of exercise, the total dose of exercise, intervention periods, and types of CHD.

**Methods:**

Following the Population Intervention Comparison Outcome Study Design (PICOS) principle, a comprehensive search of the PubMed, Web of Science, Embase and Cochrane Library databases was conducted for randomized controlled trials (RCTs) evaluating the intervention effects of aerobic exercise on cardiopulmonary function in postoperative CHD patients until December 2023. This meta-analysis and publication bias tests were conducted using Stata 17.0, and the mean differences (MDs) with 95% confidence intervals (CIs) were used as effect sizes in statistics.

**Results:**

A total of 15 RCTs (762 cases) were included in this meta-analysis, with 407 cases in the experimental group and 355 cases in the control group. Meta-analysis showed that aerobic exercise had a positive effect on Peak VO_2_ in postoperative CHD patients (MD = 2.14, 95% CI (1.34, 2.94), *p* < 0.00001, I^2^ = 36%). The analysis of subgroups showed that intervention effects of aerobic exercise were superior to the control group when patients were >18 years old (MD = 2.53, *p* < 0.00001), ≤18 years old (MD = 1.63, *p* = 0.01), under supervision (MD = 2.23, *p* < 0.00001), unsupervised (MD = 2.06, *p* < 0.00400), performing aerobic exercise (MD = 1.87, *p* = 0.0003), performing aerobic exercise combined with resistance training (MD = 2.57, *p* < 0.00010), with a total dose of exercise ≥1440 minutes (MD = 2.45, *p* < 0.00010), with the intervention period of 10–12 weeks (MD = 2.31, *p* < 0.00001), with that >12 weeks (MD = 1.97, *p* = 0.00300), or with mixed types of CHD (MD = 2.34, *p* < 0.00001).

**Conclusions:**

This meta-analysis did not deduct points for limitations, inconsistency, indirectness, imprecision, or publication bias, so the quality of evidence was graded as high. Aerobic exercise has a significantly positive impact on improving Peak VO_2_ in postoperative CHD patients. Moreover, it was found that for patients aged 18 and above, supervised aerobic exercise combined with resistance training, implemented for 10–12 weeks with a total dose of exercise ≥1440 minutes, had a better intervention effect on Peak VO_2_. This finding provided evidence-based medicine for the exercise rehabilitation of postoperative CHD patients, and explored the optimal exercise prescription for clinical practice as well.

**Clinical Trial registration:**

Registered on INPLASY No.202440016 (https://inplasy.com).

## 1. Introduction 

Congenital heart disease (CHD), caused by abnormal fetal cardiovascular development, is one of the most common congenital abnormalities [1]. According to 2020 European Society of Cardiology (ESC) Guidelines for the Management of Adult Congenital Heart Disease issued by Association for European Paediatric and Congenital Cardiology (AEPC) and International Society for Adult Congenital Heart Disease (ISACHD), approximately 9 out of every 1000 newborns worldwide are affected by CHD [2]. Being a condition present at birth, CHD leads to impaired blood supply to tissues and organs of the body, resulting in tissue hypoxia, which greatly hampers the growth and development of affected children. Moreover, hemodynamic abnormalities will exacerbate cardiac workload, predisposing patients to malignant arrhythmias and sudden cardiac death [3]. With substantial advancements in cardiac surgery and perioperative management techniques, approximately [1] 90% of CHD patients can survive to adolescence and adulthood through surgical intervention. Nevertheless, postoperative patients with CHD often encounter long-term issues such as hypoxia and reduced exercise endurance [4, 5]. These challenges directly impact patients’ oxygen supply capacity and exercise endurance, posing an urgent need for effective strategies to enhance postoperative cardiopulmonary function of CHD patients.

In the United States and Europe, the guidelines for CHD patients suggest that moderate and sustained aerobic exercise will enhance the contraction and relaxation abilities of the myocardium, promote blood circulation, increase coronary blood flow, and elevate functional capacity of the heart [6, 7]. Studies have found a range of positive effects during aerobic exercise, such as increased respiratory rate and depth, expanded lung capacity, and improved endurance, all of which can enhance the function of postoperative CHD patients, thereby improving their quality of life [8, 9, 10].

Cardiopulmonary Exercise Test (CPET) serves as a cornerstone for health evaluation and exercise prescription, and is deemed as the “gold standard” for diagnosing CHD patients’ cardiopulmonary functions [11]. Relevant indicators such as the cardiorespiratory reserve function and exercise endurance of CHD patients can be assessed by CPET. Peak oxygen consumption (VO_2_) represents the oxygen uptake when CHD patients reach their maximum exercise load during CPET, reflecting the body’s maximal aerobic metabolism and cardiorespiratory reserve capacity. As a result, it is the optimal indicator for assessing aerobic metabolic capacity. The prognostic significance of Peak VO_2_ for mortality and morbidity rates in CHD patients has been proven [12, 13, 14, 15]. Multiple studies have shown that there is an inverse relationship between Peak VO_2_ and the risk of cardiovascular events, and a direct correlation between Peak VO_2_ and cardiopulmonary functions in CHD patients [11, 16, 17]. Laukkanen* et al*. (2016) [18] demonstrated through their studies that for every increase of 1 mL/kg/min in Peak VO_2_, there is a 9% reduction in relative risk of all-cause mortality (hazard ratio = 0.91; 95% CI, 0.87–0.95), emphasizing the importance of maintaining good cardiopulmonary function. Study results from Opotowsky *et al*. (2018) [19, 20, 21, 22, 23, 24, 25, 26, 27, 28, 29] indicated that aerobic exercise can increase Peak VO_2_ in postoperative CHD patients. An RCT by Westhoff-Bleck *et al*. (2013) [26] showed that exercise for 24 weeks at a heart rate of 110.3 ± 9.7 beats per minute resulted in an increase in Peak VO_2_ (1.8 ± 2.3 mL/kg/min; +7.7%). Winter *et al*. (2012) [27] found significant changes in Peak VO_2_ after 10 weeks of aerobic exercise.

A review of prior research reveals inconsistent findings regarding the effect of aerobic exercise on Peak VO_2_ in postoperative CHD patients. The meta-analysis results by Xu C *et al*. (2020) [30] indicated that exercise has no significant impact on Peak VO_2_, a conclusion also reached by Klausen *et al*. (2016) [31]. However, studies by Opotowsky *et al*. (2018) [19, 20, 21, 22, 23, 24, 25, 26, 27, 28, 29] suggested that aerobic exercise provides substantial benefits for Peak VO_2_ in postoperative CHD patients. The disparities in these findings may be due to the small sample sizes in each experiment and remarkable age differences among participants in these studies. Furthermore, we also found that previous studies have not clarified whether different exercise elements have an effect on Peak VO_2_ in postoperative CHD patients. Thus, this study, harnessing the methodology of evidence-based medicine, aims to systematically evaluate and analyze whether aerobic exercise can effectively improve Peak VO_2_ in CHD patients. We also tried to figure out whether the effectiveness is influenced by factors such as patient age, modes of supervision, types of exercise, and the types of CHD. Moreover, this study observed whether the duration, frequency, and intervention period of aerobic exercise present a dose-response effect for improving CHD patients’ condition. In conclusion, this study aims to identify the potential dose-response effect of aerobic exercise on Peak VO_2_ in postoperative CHD patients, ascertain the optimal exercise regimen for enhancing Peak VO_2_ in postoperative CHD patients, and provide evidence-based clinical support.

## 2. Materials and Methods

Regarding the selection and utilization of research methods, this study adhered to the PRISMA writing guidelines for meta-analysis [32], and has been registered on INPLASY No.202440016 (https://inplasy.com).

### 2.1 The Research Framework

The research framework is based on 2020 ESC Guidelines for the management of adult congenital heart disease [2] issued by AEPC and ISACHD. It involves an analysis of patient information including age, modes of supervision, types of exercise, the dose of exercise, intervention periods, and types of CHD. This study aims to analyze the intervention effects of exercise on the cardiorespiratory function and exercise endurance of postoperative CHD patients by observing the changes in Peak VO_2_. Additionally, we aim to investigate potential dose-effects of aerobic exercise on the optimal intervention period, the dose of exercise, types of exercise, and modes of supervision for postoperative CHD patients. The Population Intervention Comparison Outcome Study Design (PICOS) framework for this systematic review is presented in Table 1.

**Table 1.** **PICOS framework for intervention effects of exercise on cardiorespiratory function and exercise endurance in CHD patients**.

PICOS	Inclusion criteria
Population	Patients diagnosed with CHD, excluding those with conditions such as pregnancy or history of sudden death, and those with abnormal exercise test results.
Intervention	The experimental group undergoes exercise training as an intervention. In addition to routine care, this includes aerobic exercise, resistance training, or unsupervised home-based exercise through electronic health education.
Comparison	The control group undergoes non-exercise interventions, including routine care and health education.
Outcome	Peak VO_2_
Study design	Randomized controlled trials

CHD, congenital heart disease; PICOS, Population Intervention Comparison Outcome Study Design; VO_2_, oxygen consumption.

### 2.2 Search Strategies

Two retrieval personnel searched PubMed, Embase, Web of Science, and Cochrane databases respectively to collect randomized controlled trials (RCTs) about the effects of aerobic exercise on cardiopulmonary function and exercise endurance in postoperative CHD patients. The retrieval period extended from the establishment of each database to December 31, 2023. Additionally, manual searches of previously written reviews were conducted, included in relevant literature to acquire full-text articles. The literature was searched using the following words “Heart Defects, Congenital”[Mesh] OR “congenital heart disease”[Title/Abstract] OR “atrial septal defect”[Title/Abstract] OR “ventricular septal defect”[Title/Abstract] OR “cardiac function”[Title/Abstract] OR “pulmonary hypertension”[Title/Abstract] AND “Exercise”[Mesh] OR “motion”[Title/Abstract] OR “movement”[Title/Abstract] OR “sport”[Title/Abstract] OR “physical activity”[Title/Abstract] OR “rehabilitation training”[Title/Abstract] OR “high intensity interval training”[Title/Abstract] OR “moderate intensity exercise”[Title/Abstract] OR “aerobic training”[Title/Abstract] OR “resistance exercise”[Title/Abstract] AND “Cardiorespiratory function”[Mesh] OR “exercise endurance”[Title/Abstract] OR “peak oxygen uptake”[Title/Abstract] AND “randomized controlled trial”[Publication Type] (Take PubMed as an example).

### 2.3 Inclusion and Exclusion Criteria

#### 2.3.1 Inclusion Criteria

(1) The subjects included in study were diagnosed as postoperative CHD patients by the hospital. (2) The experimental group took aerobic exercise as an intervention in addition to routine postoperative care, with the intervention period being ≥10 weeks. (3) The control group skipped exercise interventions and only adopted routine postoperative care. (4) The primary outcome was Peak VO_2_. (5) All included studies were RCTs.

#### 2.3.2 Exclusion Criteria

(1) Patients with contraindications for exercise, conditions such as pregnancy or history of sudden death, and those with abnormal exercise test results. Exercise contraindications also include patients with acute illnesses such as acute heart, liver, gallbladder, pancreas, stomach, intestine, and kidney diseases, early-stage viral myocarditis, acute viral hepatitis, acute phase of pulmonary tuberculosis, etc.; patients with hemorrhagic diseases such as leukemia, hemophilia, thrombocytopenic purpura, etc.; patients with malignant tumor metastasis; and patients with coronary artery disease, etc. (2) Duplicated publications. (3) Unclear experimental data descriptions, inconsistent baselines, lack of pre-test data, and no response from authors, making it impossible to calculate or extract data. (4) Inappropriate interventions or mismatched outcomes.

### 2.4 Study Celection, Data Extraction, and Quality Assessment

#### 2.4.1 Study Selection and Data Extraction

After retrieving the relevant literature, they would be further deduplicated in Endnote. Two researchers selected studies and extracted data independently in a double-blind trial. Extracted data were inputted into RevMan 5.4.1 (The Cochrane Collaboration, 11-13 Cavendish Square, London W1G 0AN, UK), and their accuracy was double-checked. In case of discrepancies, a third researcher would be consulted to decide whether to include the data. Extracted data included the first author’s name, publication year, publication country, baseline information of the study subjects (age, gender, and stages of recovery), interventions and outcomes.

#### 2.4.2 Quality Assessment

The methodological quality of included studies was evaluated using the Physiotherapy Evidence Database (PEDro) scale [33], which includes 10 items: “random allocation” “concealed allocation” “similarity at baseline” “subject blinding” “therapist blinding” “assessor blinding” “>85% follow up” “intention-to-treat analysis” “between-group statistical comparison” and “point and variability measures”. One point was awarded for meeting a criterion, and zero points for not meeting it. The total score was 10 points, with <4 points indicating low quality, 4–5 points indicating moderate quality, 6–8 points indicating good quality, and 9–10 points indicating high quality. Only studies of moderate quality or above were included in this study.

Evidence for the quality of outcomes was evaluated using the GRADEpro evidence rating system, which categorizes evidence quality as high, moderate, low, or very low. Quality assessment was conducted by two researchers respectively, and in case of discrepancies, a third researcher would be involved in the discussion until a consensus was reached.

### 2.5 Data Processing

RevMan 5.4.1 software was used for heterogeneity assessment of all outcomes in the included studies. The sample sizes as well as the mean and standard difference of the improvement values before and after interventions were assessed. The included outcomes were all continuous variables. For outcomes with the same measurement method and unit, mean difference (MD) was used, and for those with different measurement methods or units, the standard mean difference (SMD) was used. We used a threshold of *p* less than 0.05 and $I^{2}$ greater than 50% to represent heterogeneity for studies, and a random-effects model would be employed. Conversely, if there was no significant heterogeneity among studies (*p* ≥ 0.05 or I^2^ ≤ 50%), a fixed-effects model would be used. Through subgroup analysis, we divided the study sample into different subgroups based on specific variables (such as age, gender, stage of recovery., etc.) and conducted independent statistical analyses for each subgroup to explore and explain the differences in results among different subgroups. This helps to reveal the heterogeneity of the study results, indicating that different subgroups may respond differently to interventions or exposure factors. The process of subgroup analysis included: defining subgroups (the classifications were based on previous research literature), data segmentation, independent analysis, result comparison, and result interpretation. Sensitivity analysis involved changing key assumptions or methods during the analysis process and re-analyzing the data to test whether the original results are influenced by specific analysis choices and to evaluate the robustness of the study results. The process of sensitivity analysis included: identifying key assumptions, changing assumptions, re-analyzing, comparing results, and validating conclusions. The outcomes of our meta-analysis were presented with a 95% CI. Publication bias and heterogeneity tests were conducted using Stata 17.0 (StataCorp, College Station, TX, USA).

## 3. Results

### 3.1 Literature Search Results

**Fig. 1.**
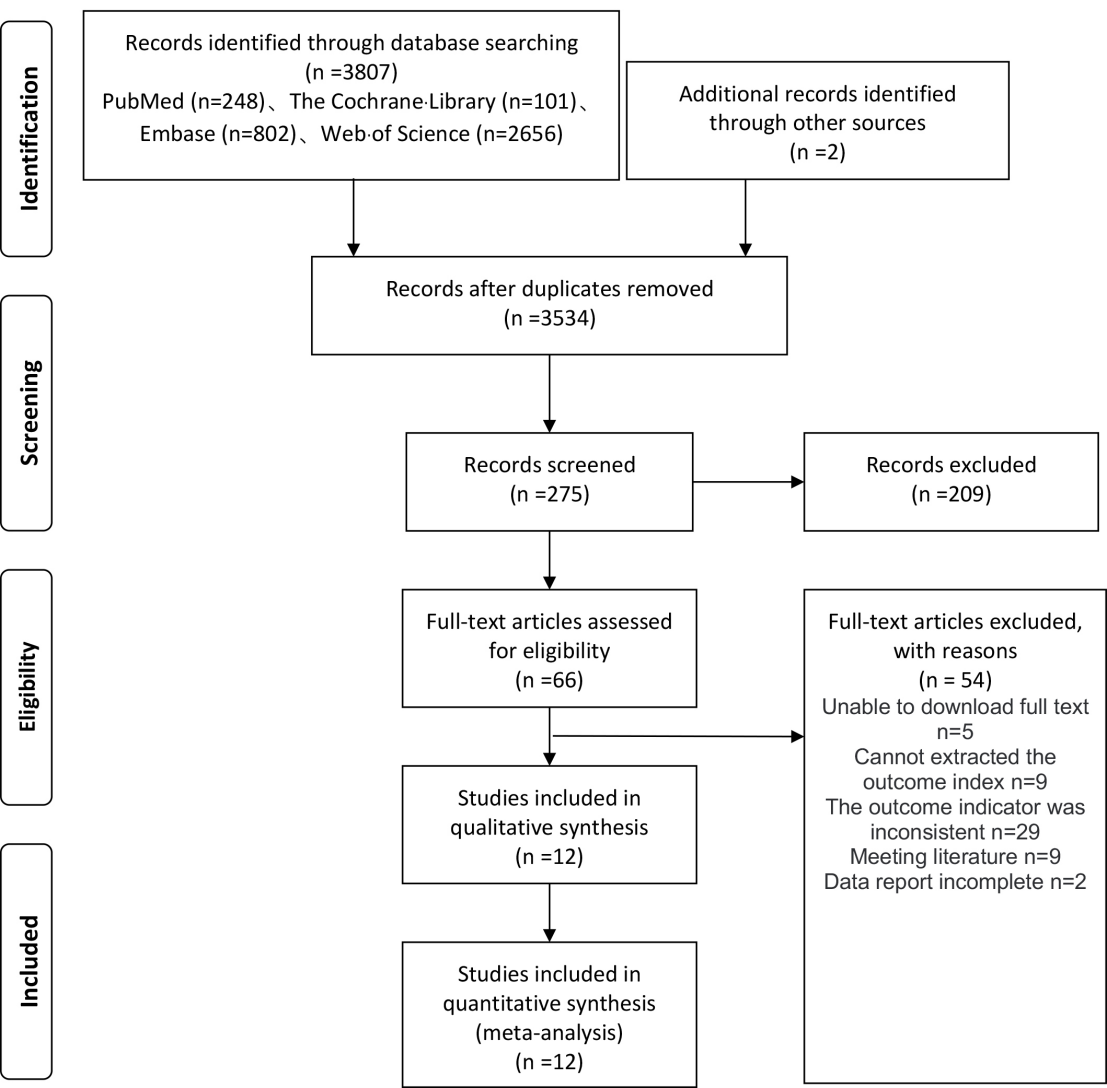
**Process of Study selection**.

A total of 3807 relevant pieces of literature were identified through searches in PubMed, Embase, Web of Science, and Cochrane databases. Additionally, 2 articles were manually retrieved from other sources. After removing duplicates and preliminary screening based on titles and abstracts, 66 articles were selected. Following a full-text assessment and exclusion of articles that failed to meet the eligibility, a final set of 12 articles were included, comprising 15 RCTs for our meta-analysis (Fig. 1).

### 3.2 Basic Characteristics of Included Studies

Table 2 (Ref. [19, 20, 21, 22, 23, 24, 25, 26, 27, 28, 29, 31]) presents the basic information of included studies, comprising 12 articles [19, 20, 21, 22, 23, 24, 25, 26, 27, 28, 29, 31] with 15 studies (762 participants). The subjects were all postoperative CHD patients with ages ranging from 8 to 43 years old. The interventions all involved aerobic exercise with exercise frequencies ranging from 2 to 5 times per week and intervention periods ranging from 10 to 52 weeks.

**Table 2.** **Basic characteristics of included studies**.

Study	Country	Location	Sample size (T/C)	Age (year) (T/C)	Intervention (T/C)	Intensity of exercise interventions	Frequency (times/week)	Period (week)	Dose (min)	Super-vison (Y/N)	Outcome [mL/kg/min]
Opotowsky *et al*. 2018 [19]	America	Rehabilitation center	28 (13/15)	47.5 ± 9.0/35.7 ± 11.9	②/③		2	12	1440	Y	Peak VO_2_
Sandberg* et al*. 2018 [20]	Swedan	Home	23 (13/10)	31.1 ± 7.2/26.3 ± 9.4	①/③	THR75%–80%	3	12	1116	N	Peak VO_2_
Morrison* et al*. 2013 [21]	Northern Ireland	Home and laboratory	143 (72/71)	15.24/15.89	①/③			24		Y	Peak VO_2_
Ávila* et al*. 2016 [22]	Canada	Institute and activity center	17 (13/4)	35 ± 11.3/34 ± 14.5	②/③	HR_max_70%–80%	2	12		Y	Peak VO_2_
Therrien* et al*. 2003 [23]	Canada	Rehabilitation center	17 (9/8)	35.0 ± 9.5/43.3 ± 7.3	①/③	Peak VO_2_60%–85%	3	12	1880	Y	Peak VO_2_
Fredriksen* et al*. 2000 [24]	Norway	Rehabilitation center and sports center	93 (55/38)	12.4 ± 1.51	①/③	HR_max_65%–80%	2	20		Y	Peak VO_2_
Klausen* et al*. 2016 [31]	Denmark	Home	158 (81/77)	13–16	①/③			52		N	Peak VO_2_
Rhodes* et al*. 2006 [25]	America	Laboratory	33 (15/18)	11.9 ± 2.2/12.1 ± 2.5	②/③		2	12	1440	Y	Peak VO_2_
Westhoff-Bleck* et al*. 2013 [26]	Germany	Home	48 (24/24)	29.9 ± 3.1/28.6 ± 3.1	①/③	Peak VO_2_50%	3–5	24	2550	N	Peak VO_2_
Winter* et al*. 2012 [27]	The Netherlands	Home	54 (28/26)	31 ± 10/34 ± 11	①/③	HR_max_75%–90%	3	10	1260	Y	Peak VO_2_
Duppen* et al*. 2015 [28]	The Netherlands	Hospital or rehabilitation center	90 (53/37)	15 ± 3	①/③	RHR60%–70%	2–3	12	1800	Y	Peak VO_2_
Novaković* et al*. 2018 [29]	Slovenia	Hospital or rehabilitation center	27 (18/9)	38.5 ± 8.7	①/③	HR_max_50%–80%	2–5	12	1260	N	Peak VO_2_

Notes: T, treatment group; C, control group; Y, yes; N, no; Intervention, ① Aerobic exercise; ② Aerobic exercise combined with resistance training; ③ Routine care; THR, training heart rate; HR_max_, maximum heart rate; RHR, resting heart rate; VO_2_, oxygen consumption.

### 3.3 Quality Assessment of Included Studies

All 12 articles included in this study employed an RCT or quasi-RCT design. Additionally, they all met the criteria of “similarity at baseline” “intention-to-treat analysis” “between-group statistical comparison” and “point and variability measures”. Additionally, 11 studies employed “random allocation”; 4 studies met the criterion of “concealed allocation”; 5 studies met the criterion of “subject blinding”; 3 studies met the criterion of “therapist blinding”; 2 studies met the criterion of “assessor blinding”; and 9 studies met the criterion of “>85% follow up”. The PEDro scores ranged from 6 to 8 points, with an average score of 6.75 points. There were no studies which scored under 5 points, indicating overall good quality of the included studies, as shown in Table 3 (Ref. [19, 20, 21, 22, 23, 24, 25, 26, 27, 28, 29, 31]).

**Table 3.** **Quality assessment of included studies**.

	Random allocation	Concealed allocation	Similarity at baseline	Subject blinding	Therapist blinding	Assessor blinding	>85% follow up	Intention-to-treat analysis	Between-group statistical comparison	Point and variability measures	Total points
Opotowsky* et al*. 2018 [19]	1	0	1	0	0	0	1	1	1	1	6
Sandberg* et al*. 2018 [20]	1	1	1	1	1	1	1	1	1	1	10
Morrison* et al*. 2013 [21]	1	0	1	0	0	0	0	1	1	1	5
Ávila* et al*. 2016 [22]	1	1	1	0	0	0	1	1	1	1	7
Therrien* et al*. 2003 [23]	1	0	1	1	0	0	1	1	1	1	7
Fredriksen* et al*. 2000 [24]	0	0	1	0	0	0	1	1	1	1	5
Klausen* et al*. 2016 [31]	1	1	1	1	1	0	0	1	1	1	8
Rhodes* et al*. 2006 [25]	1	0	1	0	0	0	1	1	1	1	6
Westhoff-Bleck* et al*. 2013 [26]	1	0	1	0	0	0	0	1	1	1	5
Winter* et al*. 2012 [27]	1	1	1	0	0	0	1	1	1	1	7
Duppen* et al*. 2015 [28]	1	0	1	1	1	0	1	1	1	1	8
Novaković* et al*. 2018 [29]	1	0	1	1	0	0	1	1	1	1	7

### 3.4 Results of the Meta-Analysis

#### 3.4.1 Primary Outcome

**Fig. 2.**
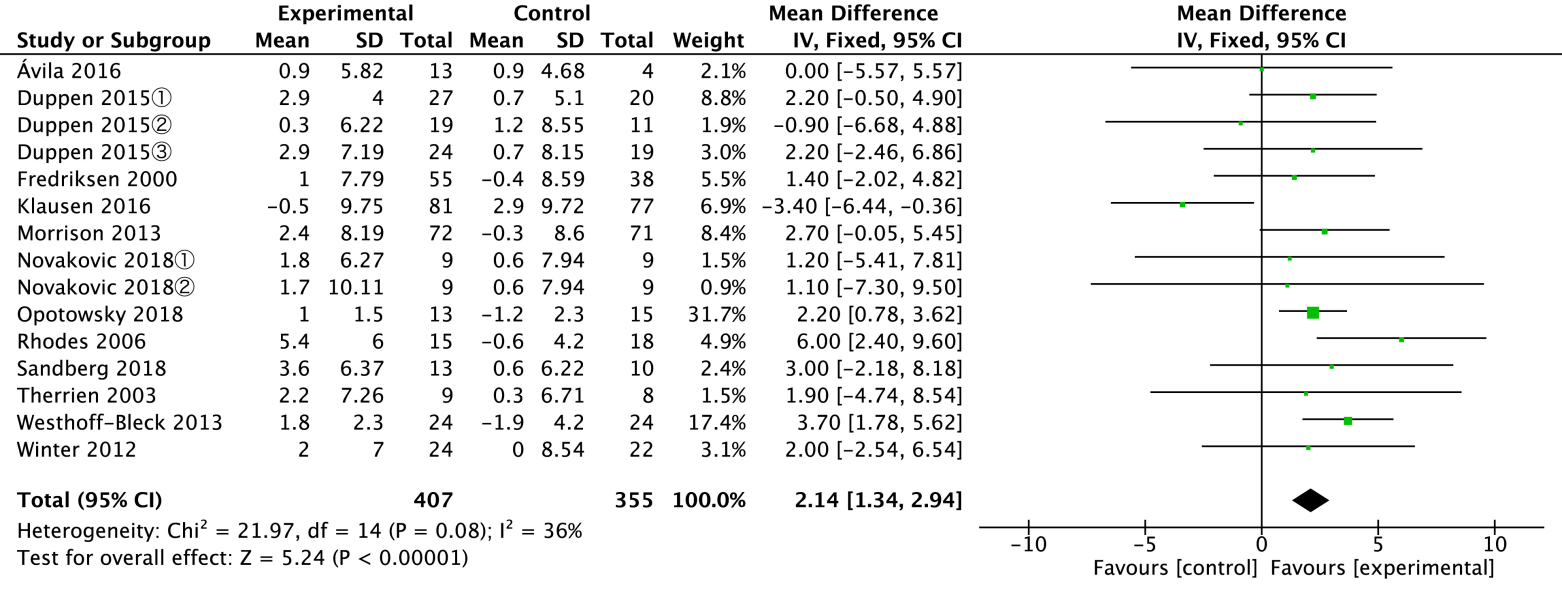
**Forest-plot: the effects of aerobic exercise on Peak VO_2_ [mL/kg/min] in postoperative CHD patients**. SD, standard deviation; IV, inverse variance; CI, confidence interval; Chi^2^, Chi-square; df, Degrees of Freedom; I^2^, I-squared; VO_2_, oxygen consumption; CHD, congenital heart disease. Duppen 2015①, Mixed patients group; Duppen 2015②, Fontan patients group; Duppen 2015③, ToF (Tetralogy of Fallot) patients group; Novakovic 2018①, Interval training group; Novakovic 2018②, Continuous training group.

The results are shown in Fig. 2, indicating that aerobic exercise effectively enhanced Peak VO_2_ in postoperative CHD patients [MD = 2.14, 95% CI (1.34, 2.94), *p* < 0.00001, I^2^ = 36%]. Since I^2^ < 50%, the heterogeneity is low, and a fixed-effects model was used.

#### 3.4.2 Heterogeneity Tests

**Fig. 3.**
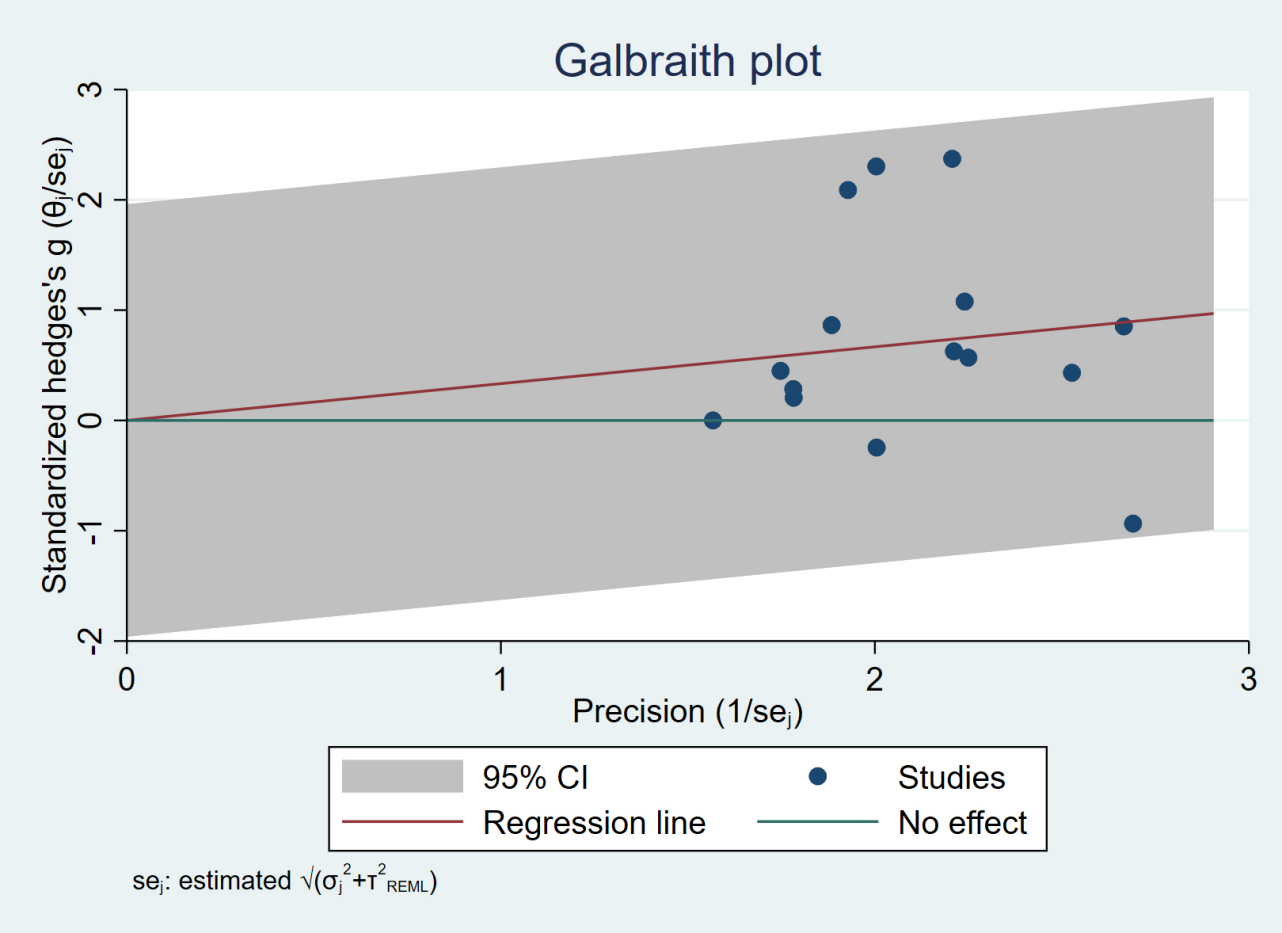
**Galbraith plot**. CI, confidence interval; se, standard error; REML, restricted maximum likelihood.

Heterogeneity tests were conducted to examine if there was significant heterogeneity among the studies. The results showed that all studies were within the range of [–2, +2], which indicated good homogeneity and a certain level of stability and reliability among the studies. See Fig. 3.

#### 3.4.3 Analyses of the Moderating Effect of Subgroups 

To explore potential sources of heterogeneity, this study analyzed outcome in subgroups, as presented in Table 4 (Ref. [19, 20, 21, 22, 23, 24, 25, 26, 27, 28, 29, 31]). The impact of aerobic exercise on Peak VO_2_ [mL/kg/min] in postoperative CHD patients may be associated with factors such as age, modes of supervision, types of exercise, the total dose of exercise (frequency of exercise time, unit: minutes), intervention periods, and the types of CHD. We categorized age into two subgroups: >18 years old and ≤18 years old; modes of supervision into supervised and unsupervised subgroups; types of exercise into aerobic exercise and aerobic exercise combined with resistance training subgroups; the total dose of exercise into <1440 minutes and ≥1440 minutes subgroups; intervention periods into 10–12 weeks and >12 weeks subgroups; the types of CHD into Tetralogy of Fallot (ToF) and mixed subgroups. Age, modes of supervision, intervention periods, and the types of CHD may be sources of heterogeneity.

**Table 4.** **Subgroups analyses results of the aerobic exercise on Peak VO_2_ [mL/kg/min] in postoperative CHD patients**.

Outcome		Number of included studies	I^2^/%	Result of meta-analysis	
				MD (95% CI)	*p*
Age	>18	8 (215) [19, 20, 22, 23, 26, 27, 29]	0	2.53 (1.5, 3.56)	<0.00001
	≤18	7 (547) [21, 24, 25, 28, 31]	62	1.63 (0.36, 2.91)	0.01000
Mode of supervision	Supervised	12 (413) [19, 20, 22, 23, 24, 25, 27, 28, 29]	0	2.23 (1.26, 3.21)	<0.00001
	Unsupervised	3 (349) [21, 26, 31]	85	2.06 (0.66, 3.46)	0.00400
Type of exercise	Aerobic exercise	10 (684) [20, 21, 23, 24, 26, 27, 28, 29, 31]	34	1.87 (0.84, 2.89)	0.00030
	Aerobic exercise combined with resistance training	3 (78) [21, 26, 31]	56	2.57 (1.28, 3.85)	<0.00010
Total dose of exercise	<1440	4 (105) [20, 27, 29]	0	2.05 (–0.8, 4.9)	0.16000
	≥1440	5 (181) [19, 25, 28]	23	2.45 (1.33, 3.58)	<0.00010
Intervention period	10–12 weeks	11 (320) [19, 20, 22, 23, 25, 27, 28, 29]	0	2.31 (1.29, 3.33)	<0.00001
	>12 weeks	4 (442) [21, 24, 26, 31]	78	1.97 (0.67, 3.26)	0.00300
Type of CHD	ToF	5 (113) [22, 23, 28, 29]	0	1.36 (–1.33, 4.05)	0.32000
	Mixed	8 (572) [19, 20, 21, 24, 25, 26, 27, 31]	61	2.34 (1.45, 3.23)	<0.00001

I^2^, I-squared; MD, mean difference; CI, confidence interval; ToF, Tetralogy of Fallot; VO_2_, oxygen consumption; CHD, congenital heart disease.

Subgroup analysis results in Table 4 (Ref. [19, 20, 21, 22, 23, 24, 25, 26, 27, 28, 29, 31]) indicate that when the total dose of exercise is <1440 minutes and the type of CHD is ToF, the difference is not statistically significant. All other indicators are statistically significant.

#### 3.4.4 Sensitivity Analysis

To investigate whether the heterogeneity among studies was caused by certain studies, this study conducted a sensitivity analysis by excluding individual studies one by one to analyze the combined effect, as shown in Table 5. After excluding the study by Klausen *et al*. (2016) [31], the combined effect was MD = –3.4, 95% CI (–6.44, –0.36), and $I^{2}$ decreased from 36% to 0%, indicating a significant reduction in heterogeneity, and the difference compared to the control group was statistically significant. Since the study by Klausen *et al*. (2016) [31] used unsupervised intervention for 52 weeks, modes of supervision and intervention periods may be sources of heterogeneity. After excluding Klausen’s study, the combined effect size MD and I^2^ all remained stable, with *p* < 0.00001, suggesting robust results, indicating that compared to the control group, aerobic exercise effectively enhanced Peak VO_2_ in postoperative CHD patients.

**Table 5.** **The combined effect of Peak VO_2_ [mL/kg/min] after excluding certain studies**.

Outcome	Excluded study	Effect size	95% CI	*p* (Combined effect)	I^2^/%
Peak VO_2_	Ávila *et al*. 2016 [22]	0	–5.57, 5.57	<0.00001	39
	Duppen* et al*. 2015① [28]	2.2	–0.50, 4.90	<0.00001	41
	Duppen* et al*. 2015② [28]	–0.9	–6.68, 4.88	<0.00001	38
	Duppen *et al*. 2015③ [28]	2.2	–2.46, 6.86	<0.00001	41
	Fredriksen *et al*. 2000 [24]	1.4	–2.02, 4.82	<0.00001	40
	Klausen *et al*. 2016 [31]	–3.4	–6.44, –0.36	<0.00001	0
	Morrison *et al*. 2013 [21]	2.7	–0.05, 5.45	<0.00001	40
	Novaković *et al*. 2018① [29]	1.2	–5.41, 7.81	<0.00001	41
	Novaković *et al*. 2018② [29]	1.1	–7.30, 9.50	<0.00001	41
	Opotowsky *et al*. 2018 [19]	2.2	0.78, 3.62	<0.00001	41
	Rhodes *et al*. 2006 [25]	6	2.40, 9.60	<0.00001	25
	Sandberg *et al*. 2018 [20]	3	–2.18, 8.18	<0.00001	41
	Therrien *et al*. 2003 [23]	1.9	–4.74, 8.54	<0.00001	41
	Westhoff-Bleck *et al*. 2013 [26]	3.7	1.78, 5.62	<0.00001	31
	Winter *et al*. 2012 [27]	2	–2.54, 6.54	<0.00001	41

CI, confidence interval; I^2^, I-squared; VO_2_, oxygen consumption. Duppen 2015①, Mixed patients group; Duppen 2015②, Fontan patients group; Duppen 2015③, ToF (Tetralogy of Fallot) patients group; Novakovic 2018①, Interval training group; Novakovic 2018②, Continuous training group.

### 3.5 Publication Bias

**Fig. 4.**
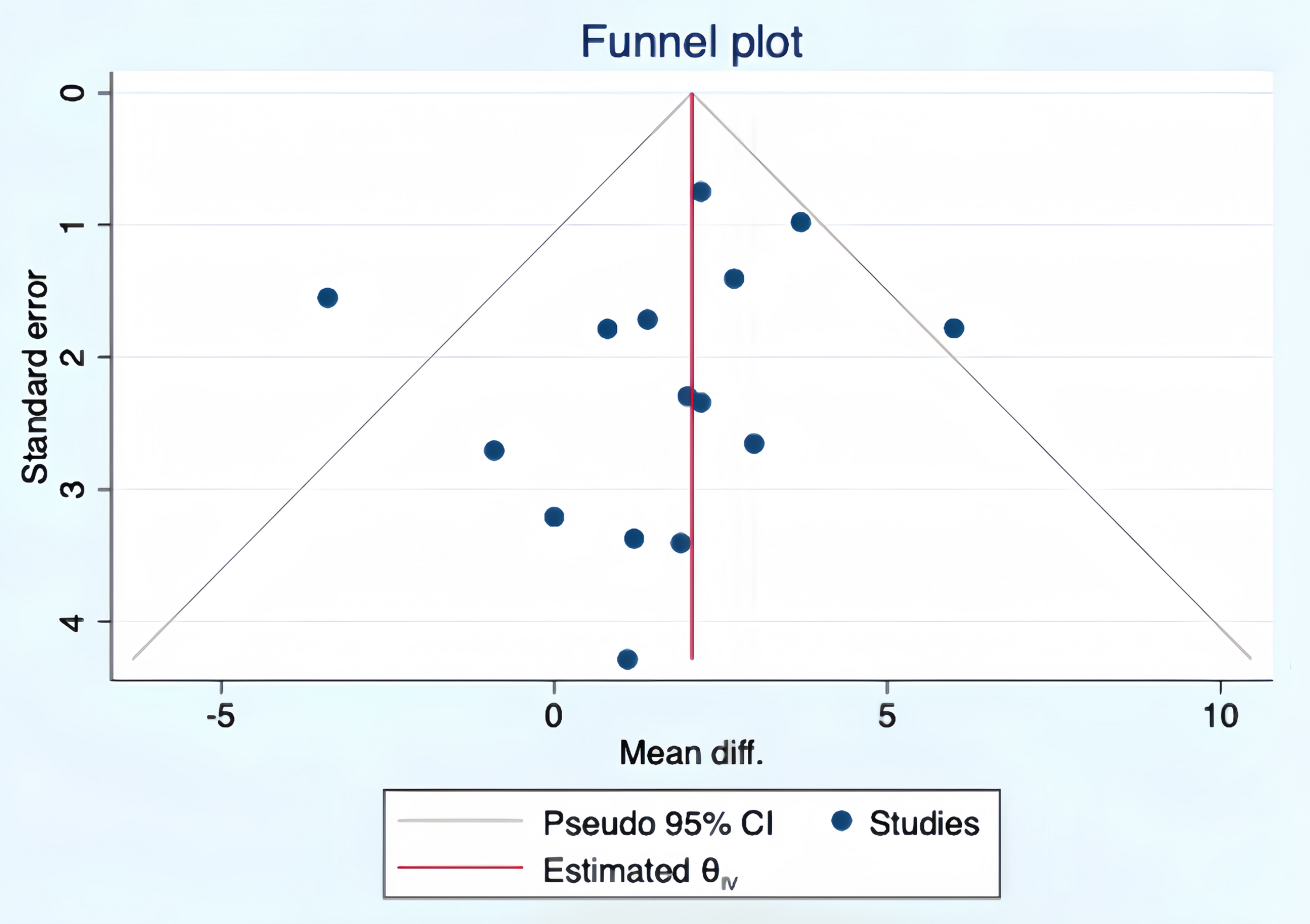
**Funnel plot of the intervention effects of aerobic exercise on Peak VO_2_ in postoperative CHD patients**. CI, confidence interval; IV, inverse variance; diff, difference; VO_2_, oxygen consumption; CHD, congenital heart disease.

The funnel plot of the intervention effects of aerobic exercise on Peak VO_2_ in postoperative CHD patients shows symmetrical distribution. Egger’s Test yielded a result of t = –0.64, *p* > |t| = 0.5338, indicating no publication bias in the studies, as shown in Fig. 4.

### 3.6 Quality of Evidence Assessment

According to GRADEPro, there were no deductions in terms of limitations, inconsistency, indirectness, imprecision, and publication bias of this study. Therefore, the quality of evidence was graded as high (Table 6). The above results suggested that the intervention effects of aerobic exercise on Peak VO_2_ in postoperative CHD patients is likely close to reality.

**Table 6.** **Quality of evidence assessment by GRADE**.

Outcome	Included studies	Quality of evidence assessment					Quality
		Limitation	Inconsistency	Indirectness	Imprecision	Publication bias	
Peak VO_2_	15	Not serious	Not serious	Not serious	Not serious	Not serious	High

GRADE, Grading of Recommendations, Assessment, Development and Evaluation; VO_2_, oxygen consumption.

### 3.7 Adverse Events

In the study by Sandberg *et al*. 2018 [20], one participant experienced discomfort and arrhythmia during exercise training, leading to the cessation of exercise. However, no arrhythmia was observed in subsequent exercise tests and dynamic electrocardiograms. Apart from this event, no other exercise-related adverse events occurred.

## 4. Discussion

The results of this study showed that aerobic exercise can improve Peak VO_2_ in postoperative CHD patients. A MD of 2.14 in Peak VO_2_ was observed. This change indicated that even a slight increase in Peak VO_2_ may signify a significant improvement in cardiopulmonary function and exercise capacity in CHD patients [21]. Therefore, the increase of Peak VO_2_ not only provides clinicians with a valuable indicator for assessing patient prognosis but also offers valuable guidance for developing personalized treatment plans and exercise prescriptions. The meta-analysis results of Li *et al*. 2019 [34] also supported this conclusion (MD = 1.96). Additionally, the result validated the study by Gomes-Neto *et al*. 2016 [34, 35], indicating the effectiveness of aerobic exercise intervention on Peak VO_2_ in child, adolescent, or adult postoperative CHD patients. Meyer *et al*. 2020 [36] suggested that unsupervised home-based aerobic exercise can improve Peak VO_2_ in postoperative CHD patients, aligning with the results of this study. Although the mechanism by which exercise increases Peak VO_2_ remains unclear, exercise has been proven to improve peripheral muscle function [37], enhance autonomic nervous system regulation, increase vagal activity, inhibit sympathetic activation, and reduce levels of angiotensin and renin, thereby improving cardiopulmonary function [34].

Our study included a total of 15 RCTs (762 patients) for a systematic review and analysis of the intervention effects of aerobic exercise on Peak VO_2_ in postoperative CHD patients. Included studies were quality-assessed by the PEDro scale, with an average score of 6.75 points. There were no low-quality studies found, indicating satisfactory quality of the overall included studies. There appeared to be no noticeable publication bias. I^2^ = 36%, after subgroup analysis, it was found that the patients’ age, modes of supervision, types of exercise, the total dose of exercise, intervention periods, and types of CHD might be sources of heterogeneity; sensitivity analysis revealed that intervention periods and modes of supervision could be sources of heterogeneity. The quality of evidence evaluation had no deductions for limitations, inconsistency, indirectness, imprecision and publication bias. Overall, the intervention effects of aerobic exercise on Peak VO_2_ in postoperative CHD patients were classified under high quality evidence. In addition, this study has certain limitations. There was certain heterogeneity among exercises with different types, frequencies, intensities, durations, and periods, which may affect the overall effect size and subgroup analyses. Besides, it was difficult to implement complete blinding in exercise interventions, and there was diversity in the patients’ conditions and stages of recovery.

In this study, it was found that for adult patients aged 18 and above, supervised aerobic exercise combined with resistance training, implemented for 10–12 weeks with a total dose of exercise ≥1440 minutes, helps to significantly improve Peak VO_2_. This may be attributed to the differing cardiovascular responses between adults and children. The smaller size of children’s hearts results in lower venous return and consequently lower cardiac output, leading to a lower Peak VO_2_ compared with adults, thereby resulting in less significant variations in Peak VO_2_ [38]. The intervention period of 10–12 weeks with a minimum exercise dosage of 1440 minutes yielded the best outcomes, possibly due to the more pronounced immediate effects of aerobic exercise. Besides, with the increase of total treatment duration within the same intervention period, the increased treatment volume reaches a threshold that beneficially impacted cardiopulmonary function. Regarding the mode of supervision, on-site supervision by trainers proved most effective, followed by unsupervised home-based exercise accompanied with consultation, encouragement, and electronic applications, which also contributed to the rehabilitation of cardiopulmonary function in patients. There is research indicating that home-based exercise interventions are safe and feasible, representing an effective alternative to supervised cardiac rehabilitation [36]. Aerobic exercise combined with resistance training can slightly improve cardiopulmonary health by mechanisms such as increasing muscle strength, thus improving oxidative enzymes and increasing type II muscle fibers [39].

The results of this study also demonstrated that there was no significant difference in intervention effects of aerobic exercise on the ToF subgroup. Williams *et al*. (2020) [40], in their meta-analysis, arrived at the same conclusion. However, among included studies, results of 4 RCTs by Ávila *et al*. (2016) [22, 23, 28, 29] all indicated a significant intervention effect of aerobic exercise on Peak VO_2_ in postoperative ToF patients, with only Novaković et al. (2018) [29] expressing disagreement. The reason for the non-significant intervention effect of aerobic exercise on the ToF subgroup in this study may be attributed to insufficient sample size, necessitating further high-quality RCTs for clarification.

## 5. Conclusions

In summary, aerobic exercise could significantly improve Peak VO_2_ in postoperative CHD patients. For patients aged 18 and above, a 10–12 weeks supervised intervention integrating aerobic exercise with resistance training, with a total exercise dosage of at least 1440 minutes, yielded better results. Current evidence of included studies suggests that aerobic exercise is significant and safe for enhancing Peak VO_2_ in postoperative CHD patients. Despite certain limitations, our study provided evidence-based medicine for the exercise rehabilitation of postoperative CHD patients. Finally, we look forward to further research in this area to explore the intervention effects and mechanisms of exercise on postoperative CHD patients with different conditions and at different stages of recovery.

## Data Availability

The datasets used and/or analyzed during the current study are available from the corresponding author on reasonable request.

## Abbreviations

CHD, congenital heart disease; RCTs, randomized controlled trials; MD, mean difference; SMD, standard mean difference; CI, confidence interval; AEPC, Association for European Paediatric and Congenital Cardiology; ISACHD, International Society for Adult Congenital Heart Disease; ToF, Tetralogy of Fallot; CPET, Cardiopulmonary Exercise Test.

## Supplementary Material

Supplementary material associated with this article can be found, in the online version, at https://doi.org/10.31083/j.rcm2508296.
